# Bio-Epoxy Resins Based on Lignin and Tannic Acids as Wood Adhesives—Characterization and Bonding Properties

**DOI:** 10.3390/polym16182602

**Published:** 2024-09-14

**Authors:** Ivana Gavrilović-Grmuša, Milica Rančić, Tamara Tešić, Stevan Stupar, Milena Milošević, Jelena Gržetić

**Affiliations:** 1 Faculty of Forestry, University of Belgrade, 11030 Belgrade, Serbia; milica.rancic@sfb.bg.ac.rs (M.R.); student.tamaratesic2262002@sfb.bg.ac.rs (T.T.); 2 Military Technical Institute, Ratka Resanovića 1, 11000 Belgrade, Serbia; stevan.stupar13@gmail.com (S.S.); jrusmirovic@tmf.bg.ac.rs (J.G.); 3 Institute of Chemistry, Technology and Metallurgy, National Institute of the Republic of Serbia, University of Belgrade, 11000 Belgrade, Serbia; milena.milosevic@ihtm.bg.ac.rs

**Keywords:** tannic acid, lignin, epoxy lignin, bio-hardener, bio-epoxides, tensile shear strength

## Abstract

The possibility of producing and designing bio-epoxides based on the natural polyphenol lignin/epoxidized lignin and tannic acids for application as wood adhesives is presented in this work. Lignin and tannic acids contain numerous reactive hydroxyl phenolic moieties capable of being efficiently involved in the reaction with commercial epoxy resins as a substitute for commercial, non-environmentally friendly, toxic amine-based hardeners. Furthermore, lignin was epoxidized in order to obtain an epoxy lignin that can be a replacement for diglycidyl ether bisphenol A (DGEBA). Cross-linking of bio-epoxy epoxides was investigated via FTIR spectroscopy and their prospects for wood adhesive application were evaluated. This study determined that the curing reaction of epoxy resin can be conducted using lignin/epoxy lignin or tannic acid. Tensile shear strength testing results showed that lignin and tannic acid can effectively replace amine hardeners in epoxy resins. Examination of the failure of the samples showed that all samples had a 100% fracture through the wood. All samples of bio-epoxy adhesives displayed significant tensile shear strength in the range of 5.84–10.87 MPa. This study presents an innovative approach to creating novel cross-linked networks of eco-friendly and high-performance wood bio-adhesives.

## 1. Introduction

The increasing concern around environmental issues such as fossil resources and global warming has brought wood and innovative wood products to the center of interest due to their beneficial impact on lowering greenhouse gas (GHG) emissions and carbon footprints [1]. However, the possibility of the wood being used for new and demanding construction tasks depends to a great extent on adhesive technologies. The crucial issue must include switching to environmentally friendly adhesives, lower carbon dioxide emissions, and altogether greener solutions. The environmental friendliness of engineered wood products—as the only known renewable and sustainable construction material—is impacted by the adhesives used, usually synthesized and produced from oil-based raw materials, e.g., petroleum and natural gas [2].

Environmental legislation has also directed attention to a green design approach and producing bio-based adhesives from raw materials for a more sustainable and aware society. Therefore, formulating an environmentally friendly adhesive from renewable resources represents the central issue in meeting today’s green demands of the woodworking industry [3,4]. For structural applications, it is of utmost importance to make the adhesive joint as strong as, or even stronger than, the wood itself [5]. However, an effective wood adhesive requires a uniform molecular weight distribution, suitable viscosity, good water resistance, and a stable bond performance [6]. Conventional wood adhesives are produced using four major synthetic thermosetting resins: phenol-formaldehyde (PF), urea-formaldehyde (UF), melamine formaldehyde (MF), and polymeric diphenylmethane diisocyanate (pMDI) resin [7].

Epoxy resins (ER) are thermosetting polymers with a broad spectrum of characteristics related to their structure and applications, such as building construction materials (e.g., coatings, adhesives, and advanced composites). ERs, as effective chemical cross-linkers, exhibit excellent properties, including good bonding performance [5] and thermal and chemical stability [8]. The most common type of ER is diglycidyl ether bisphenol A (DGEBA), which is very reactive to many curing agents such as amine hardeners (AH), isocyanates, or acid anhydrides [9], which are usually non-environmentally friendly. Replacing these curing agents (hardeners) with more environment-friendly types is a tremendous technological challenge for many researchers today [8,10].

It is crucial to find bio/raw materials that can be used for the ER preparation without extensive modification/functionalization [11]. Bio-based thermoset polymers produced from natural resources such as sugars, polysaccharides, vegetable oils (VOs), lignins, lipids, proteins, and other monomers have attracted great attention in both research and industrial applications [12,13,14]. The conversion of waste biomass and industrial by-products such as tannins and lignins into specialty added-value chemicals and materials has not only directed the development of an efficient and environmentally benign technology and consumer products but has also coincidently revealed the problem of agricultural and forest waste [15,16,17,18,19,20]. In this sense, the main emphasis is on natural phenolic compounds, tannins, and lignins, which have been proposed as promising solutions for replacing the commercial/synthetic ER or curing hardeners.

Tannin-based resins have been used as a replacement for the phenolic resins applied in the production of wood-based panels due to their high moisture resistance and decreased formaldehyde emissions. Tannins can react with formaldehyde and form hardened and cross-linked moieties by forming oxymethylene and methylene linkages which are stable against hydrolysis [4]. Due to the reactivity of the tannin hydroxyl groups attached to the aromatic ring, other substances can react with them in order to obtain materials with improved physical and chemical properties [21,22]. The application of tannins in materials/composite preparation has been investigated, and their efficiency in improving the corrosion, mechanical, and thermal performances of the material is demonstrated [22,23,24]. Moreover, for tannin applications in materials preparation, the type of tannin is significant. There are two main categories of tannins: hydrolyzable and non-hydrolyzable (or condensed tannins) [25]. Hydrolyzable tannins, including tannic acids (derivatives), are more suitable for processing. Tannic acid represents a tannin derivative that consists of the gallic and hexahydroxydiphenic acid units linked via ester bonds and contains numerous reactive phenolic hydroxyl groups which can efficiently react with the epoxy groups of commercial ERs. They can be used as a substitute for commercial, non-environmentally friendly amine-based cross-linkers/hardeners [26,27]. Kim et al. studied the mechanical and flame-retardant properties of tannin-based epoxy resins, varying the tannic acid (TA) content. They found that a curing process occurs between DGEBA and TA, but that a high TA concentration in ER induces incomplete hardening due to phase separation [8].

Lignin (L), as one of the main wood constituents, represents the most abundant natural aromatic biopolymer. Although huge amounts of lignin are obtained annually because lignin is a by-product of the pulping industry, it is usually considered a low-value product. It is not reported that any lignins have been used as a sole adhesive for wood, but it represents a promising candidate due to its low cost and high availability, despite it not being as reactive as tannin [28]. There have been some attempts for lignins to be used in aminoplastic and phenolic resins [7]. Methylated lignins have replaced up to half the amount of phenol in modified phenol-formaldehyde (PF) resins in some restricted applications, mainly for producing plywood [7]. However, there have been some challenges in using lignin to replace DGEBA in ERs, such as the high polydispersity index and molecular weight, different types of hydroxy groups, and low solubility in organic solvents and water. These lignin characteristics bring about its lower reactivity towards epichlorohydrin (ECH) than DGEBA, thus resulting in resin with a lower homogeneity. Lignin can be incorporated into epoxy resin via blending with petroleum-based epoxy resin or via the epoxidation of unmodified lignin to obtain epoxy lignin (EL) [29,30,31,32]. Researchers have reported the incorporation of lignin into the epoxy polymers used in the microelectronics industry [33]. Despite several reports of lignin-based adhesive formulations, none have been commercially successful, primarily due to processing constraints at the industrial scale [15,16,17,18,34,35,36,37]. We focused on addressing these processing limitations in our design of tannin acid and lignin-based adhesives. Small concentrations of unmodified lignin can be incorporated into epoxy formulations to improve polymer mechanical properties, although its high concentrations can cause phase separation and brittle behavior. Replacing DGEBA with lignin is still challenging because it possesses a high molecular weight, different types of hydroxyl groups, and low solubility in organic solvents as well as in water. Chemical modification of lignin overcomes most drawbacks, improving its reactivity, solubility, and practical adhesion [38]. Chemically modified lignin can substitute both components of the system: epoxy resin and/or hardener.

**Figure 1:**
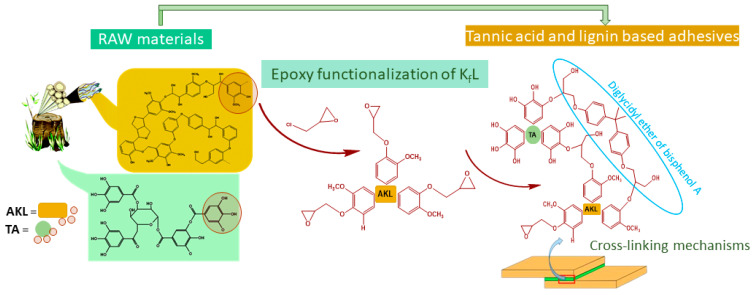
Cross-linking reactions of tannic acids (TA), Kraft lignin (AKL), and epoxy-modified lignin (EL) with ERs.

To the best of our knowledge, there has not been any study related to the preparation of epoxy resin based on natural aromatic polymers, tannic acids and lignin used as wood adhesives. Herein, a method was provided for preparing epoxy lignin and new ERs based on commercial epoxy resins with different loading of TA and/or lignin/epoxy lignin (Figure 1). This work aimed to investigate the effect of tannic acid and lignin/epoxy lignin incorporation and their loading on the adhesive properties of the epoxy resins obtained, with the aim of estimating the possibility of their potential application as wood adhesives.

## 2. Materials and Methods

### 2.1. Materials

Tannic acid (MW = 1702.20 g/mol), alkali kraft lignin (AKL), absolute ethanol, epichlorohydrin (ECH), sodium hydroxide (NaOH), *N*,*N*-dimethylformamide (DMF), and tetrabutylammonium bromide (TBAB) were purchased from Sigma-Aldrich (Steinheim, Germany). Epoxy resin (DGEBA), component A (CompA, ER), and amine hardener, the mixture of 3-aminomethyl-3,5,5-trimethylcyclohaxylamine and 2,2,4-trimethylhexan-1,6-diamine, and component B (CompB, AH) were purchased from Epoksan (Čačak, Serbia).

### 2.2. Epoxy Modification

#### 2.2.1. Epoxy Modification of Lignin

A literature review showed several methods for the epoxidation of AKL. Lignin epoxidation, i.e., its reaction with epichlorohydrin (ECH), effectively introduces epoxy groups into the lignin structure. According to the previous literature data, the optimal conditions for the synthesis were determined to be 8 h at 55 °C with a NaOH/L molar ratio of 6.3; a high product yield (99%), and a high epoxy content of ~8 were achieved [39]. Thus, in this study, the optimal reaction conditions based on the literature data were applied. Lignin epoxidation was performed using the AKL reaction with ECH at the molar ratio of lignin/NaOH = 1/1.5 wt%. Since all lignin samples showed good solubility in DMF, this was used as a co-solvent. After dissolving the AKL sample (8 g) in DMF (40 g) and stirring for 10 min at room temperature, the catalyst tetrabutylammonium bromide (TBAB; 0.8 g) was added to the lignin/DMF solution containing ECH (80 g). The reaction mixture was stirred and refluxed for 3 h at 60 °C. Subsequently, 40 wt% NaOH solution (50 mL) containing 1.2 wt% TBAB was gradually added dropwise to the cooled-down reaction mixture. The reaction continued at room temperature for 8 h when deionized (DI) water (1000 mL) was added to the reaction mixture to precipitate epoxidized lignin (EL). The excess solvent was removed by vacuum filtration and the epoxidized lignin was extracted and washed several times with DI water to remove the formed salt and unreacted ECH. Finally, the epoxidized lignin was dried in a vacuum oven at 40 °C and 76 kPa for 48 h.

#### 2.2.2. Epoxy Equivalent Weight

The epoxy equivalent weight (EEW) of the epoxy lignin was determined according to ASTM D 1652. In total, 0.4 g of sample was dissolved in 10 mL methylene chloride in a 150 mL flask. Then, 10 mL of tetraethyl ammonium bromide solution in glacial acetic acid (concentration 0.25 g/mL) was added to the flask with 6–8 drops of crystal violet indicator. The mixture was stirred and titrated with standardized 0.1 M perchloric acid solution to an endpoint transition from sharp blue to green.

### 2.3. Curing (Epoxy Adhesive Preparation)

Bio-epoxy adhesive resins were prepared by mixing commercial epoxy resin as Component A (ER) and an amine hardener as Component B (AH), or tannic acids (TA) and kraft lignin (L)/epoxy lignin (EL), according to the mass ratios shown in Table 1. The control sample was prepared by mixing component A (Comp A) and the amine hardener (Comp B). Bio-epoxy resin was obtained by combining the commercial epoxy resin with kraft lignin/epoxy lignin in percentages of 5, 10, and 15% in relation to the adhesive mixture. The curing agent was added according to the manufacturer’s procedure to achieve the ER/AH ratio of 2:1 *w*/*w*.

The resin samples were labeled according to their composition, indicating the quantity of lignin/epoxy lignin incorporated during the epoxy resin synthesis. ER-AH-L5, ER-AH-L10, and ER-AH-L15 refer to samples with 5 wt%, 10 wt%, and 15 wt% of lignin, respectively. Accordingly, ER-AH-EL5, ER-AH-EL10, and ER-AH-EL15 are the epoxidized-lignin counterparts. The other batch of bio-epoxy resins were prepared with tannic acids instead of the commercial curing agent in the ratio of commercial epoxy to tannic acids of 2:1, and then lignin/epoxy lignin was added in the appropriate quantity (5, 10, or 15%, as shown in Table 1). Tannic acids were dissolved in ethanol before mixing (2 g in 1.4 mL). The obtained mixtures were homogenized using a mechanical stirrer for 30 min.

### 2.4. FTIR Analysis

Structural characterization of the raw materials and the cured samples was determined by FTIR spectroscopy in absorbance mode using a Nicolet™ iS™ 10 FT-IR Spectrometer (Thermo Fisher SCIENTIFIC, Waltham, MA, USA) with Smart iTR™ Attenuated Total Reflectance (ATR) equipped with a single reflection diamond crystal. Sampling accessories were within a range of 400–4000 cm^–1^ at a resolution of 4 cm^–1^ and in 20 scan mode.

### 2.5. Adhesion Testing Methods

The shear strength of wood adhesives is regularly evaluated using standard testing methods [40]; the ASTM D 905 standard block shear specimens are frequently used for evaluating the adhesive bonds in solid wood materials, and the EN 205 are used for non-structural applications. These are the most commonly used standards in European wood research.

#### 2.5.1. Preparation of Wood Samples

Beech (*Fagus sylvatica*) logs were selected from a known locality and growth conditions (beech from mountain Goč). Afterwards, primary boards with the desired orientation of growth rings were cut. The sawn timber was dried in a semi-industrial conventional kiln (Nigos MC 3000, capacity 0.8 m^3^). The most homogeneous groups of testing samples were selected for further experiments. The sample joints with dimensions of 150 mm × 20 mm × 5 mm were prepared.

#### 2.5.2. Preparation of Joint Samples for the Determination of Glue-Line Quality

Testing samples were prepared by applying the resin samples with a rubber roller onto each surface of the two wood specimens to be bonded (200 g/m^2^). It was important to have the taper as low as possible, guaranteeing equal penetration conditions for all samples. The assembly was performed carefully in parallel grain directions. Five joint samples for each adhesive combination were pressed in a hydraulic press at 120 °C and 1.0 MPa for 30 min. Before the test was performed, an ammonium nitrate atmosphere was used for the conditioning of the test samples at a relative humidity of (65 ± 5)% and a temperature of (20 ± 2) °C for seven days. According to the standard procedure (SRPS EN 12765), samples fulfilled the minimum adhesive strength values for thin bond lines. Afterwards, according to standard SRPS EN 205, the tensile shear strength of the adhesive joints was determined using a hydraulic machine for the testing of mechanical properties of wood samples “Wood tester WT4”, using a measuring scope of 40 kN and a testing speed of 3 mm/min. A light microscope was used to determine the failure zone (20 mm × 10 mm) in order to determine the type of failure, the proportion of wood failure, and the thickness of the wood layer in the wood failure. Five replications were performed for each set of parameters. An analysis of variance (ANOVA) was applied to obtain centralized values and standard errors. The diverse types of failure after completion of the adhesion tests, i.e., cohesive, adhesive, and adherent failure, were considered by analyzing the surface of both specimens through high-resolution imaging.

### 2.6. SEM Analysis

The morphology of the fractures in the commercial epoxy adhesive and bio-epoxy adhesives were studied by scanning electron microscopy with Energy-Dispersive X-ray Spectroscopy using the JEOL 6610LV SEM/EDS (Jeol Ltd., Tokyo, Japan). The surface conductivity of all samples was obtained by applying gold using a Q150 ES sputtering coater (Quorum Technologies, Laughton, UK).

## 3. Results and Discussion

### 3.1. Lignin and Epoxy Lignin

**Figure 2:**
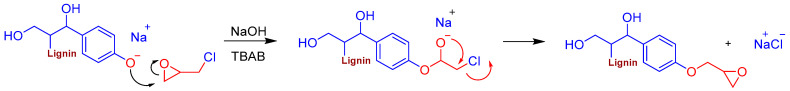
Mechanism of lignin epoxidation reaction by epichlorohydrin in alkaline conditions to obtain epoxy lignin (EL).

Lignin was successfully epoxidized under the selected reaction conditions, and a black powder-epoxy lignin was obtained. Reaction time, temperature, raw material ratio, and the quantity of alkaline solution influenced the yields, and epoxy indexes of the epoxidation reaction are presented in Figure 2. The lignin epoxidation reaction often takes 1–8 h and is usually performed using organic solvents because epichlorohydrin dissolves poorly in water. However, the intermediate product in the lignin-epoxidation reaction is highly polar (Figure 2), and the presence of water could perhaps stabilize the intermediate product and thus increase the reaction rate. The epoxidation reaction is highly exothermic, and the elevated temperature has a negative effect on the reaction. While the unmodified lignin is poorly soluble in water below pH 10, the epoxidized lignin obtained was water-soluble at a pH above 8. The introduction of the epoxy moiety and the reduction of the phenolic hydroxy groups lead to decreased intermolecular hydrogen bonding and improved water solubility. The epoxy equivalent weight (EEW) of the epoxy lignin was determined to be 4.8 mmol per gram of resin which is lower but comparable to the commercial DGEBA epoxy content (5.3 mmol/g). Hence, this synthesized epoxy lignin can be a suitable replacement for ER.

### 3.2. Structural Characterisation

#### FTIR Spectra of Epoxy Lignin and Bio-Epoxy Adhesives

**Figure 3:**
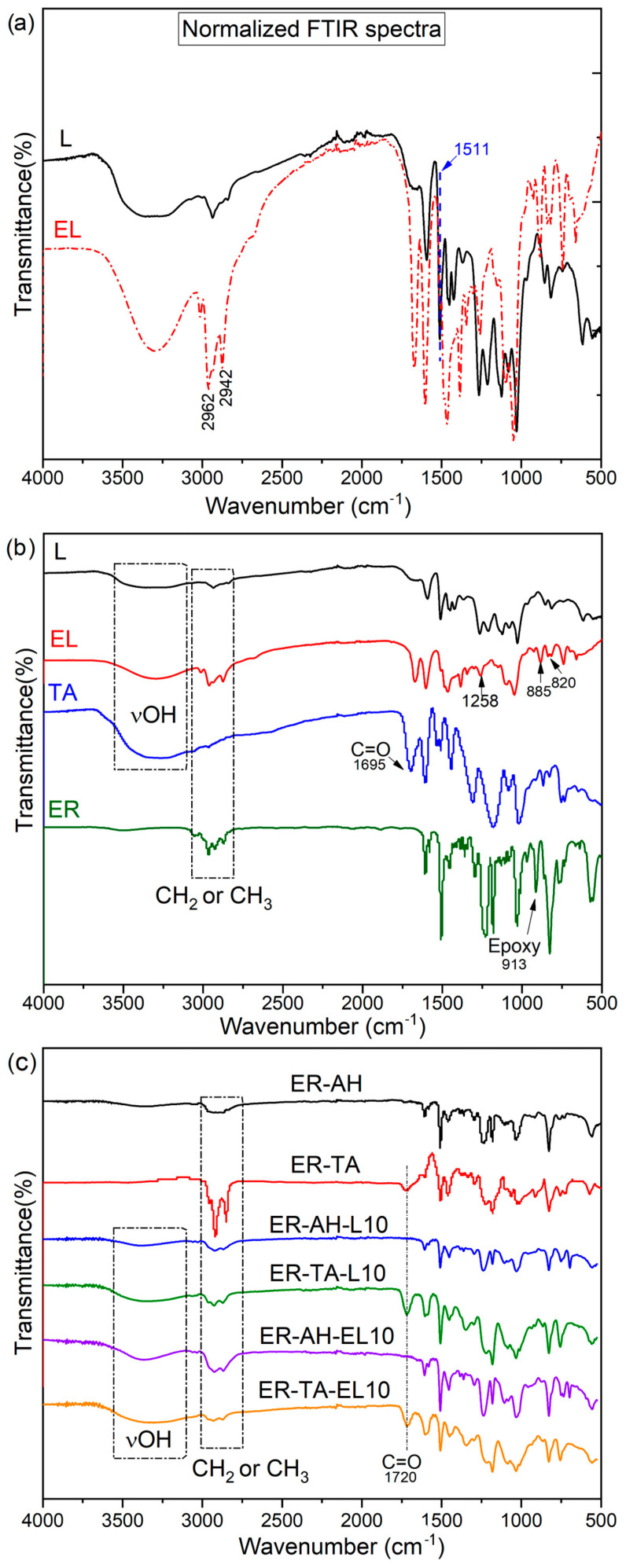
FTIR spectra of the (**a**) normalized FTIR (**b**) raw materials and (**c**) cured control epoxy and TA/L bio-epoxy adhesives.

Structural changes in the lignin surface functionality were determined using FTIR spectroscopy (Figure 3). The lignin, EL, and TA spectra were mainly characterized by broad -OH bands at 3555–3000 cm^−1^ originating from -O-H stretching vibrations of phenolic and aliphatic hydroxy groups. The intense bands in the region 2950–2850 cm^−1^ were assigned to -CH_2_/-CH_3_ asymmetrical and symmetrical stretching [34].

The broad OH band increased in the EL spectrum (Figure 3a,b). This is in accordance with previous studies in which ^31^P NMR analysis confirmed that the contents of phenolic -OH dropped sharply after epoxidation, indicating successful epoxidation. However, the amount of aliphatic OH groups increased due to incomplete epoxidation, that is, the higher temperature led to the hydrolysis of derivatized OH groups [31,41,42]. The bands below 3000 cm^−1^ (region 2950–2850 cm^−1^), originating from the methyl and methylene groups’ stretching vibrations, increased in the EL spectrum. The absorption at 1671 cm^−1^ refers to the presence of the stretching vibration of the aliphatic carbonyl group C=O which is present in lignin. The band at 1602 cm^−1^ represents a combination of -C=O and -C=C vibrations from the aromatic ring, and at 1511 cm^−1^ belongs to the aromatic C=C bonds; this band was used for normalizing the L/EL spectra [43]. The signal at 1383 cm^−1^ observed for the lignin originated from the bending vibration of the phenolic hydroxyl group, and disappeared in the spectrum of the epoxidated lignin because of the reaction between the lignin phenolic groups and epichlorohydrin. The introduction of epoxy rings into the lignin resulted in the appearance of the peaks at 1258 cm^−1,^ and in the region 885–820 cm^−1^ that differentiated them from the lignin in the FTIR spectrum (Figure 3a) [43]. The peak at 913 cm^−1^ also corresponds to the epoxy group vibration, with the highest intensity in the ER spectrum (Figure 3b).

**Figure 4:**
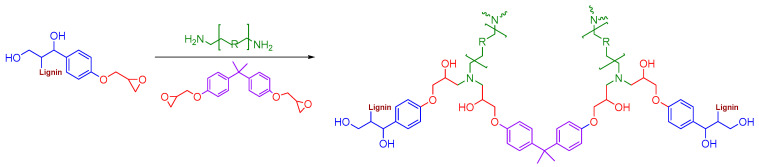
Mechanism of the crosslinking of DGEBA, epoxy lignin, and amino hardener during curing.

**Figure 5:**
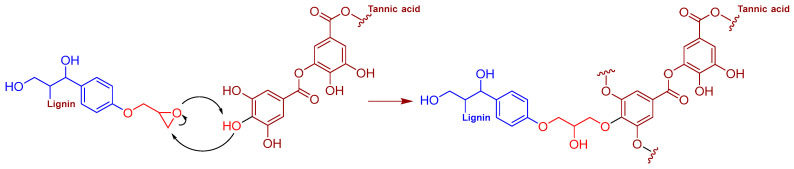
Mechanism of the crosslinking of epoxy lignin and tannic acid during curing.

The FTIR analysis was also used to confirm the structure and curing process of the TA/L-based bio-epoxides; the mechanisms underlying the crosslinking of DGEBA, epoxy lignin, and tannic acid during curing are presented in Figure 4 and Figure 5. The broad bands at 3555–3000 cm^−1^ in the L, EL, and TA spectra related to O-H stretching vibrations can be also observed in the FTIR spectra of all synthesized TA/L-based bio-adhesives (Figure 3c). The FTIR spectra of the commercial epoxy resin (ER), cured control sample (ER-AH), ER-TA, and lignin-based bio-adhesives (ER-AH-L10, ER-TA-L10, ER-AH-EL10, and ER-TA-EL10) are presented in Figure 3b. The strong signals around 1610 cm^−1^ and 1500–1510 cm^−1^ can be ascribed to the C=C–C vibrations of TA aromatic rings, ER, and cured bio-epoxides. The bands in the region 1050–1300 cm^−1^ originated from the C-O vibrations of phenolic hydroxy groups. The C=O ester group band, presented in the FTIR spectra of TA and L (Figure 3b), was shifted to the higher wavenumber length (1720 cm^−1^) in the spectra of the cured TA/L bio-epoxides (Figure 3b) [44]. The vibration frequency of the C=O ester band shifts to the higher wavenumber (frequency) because crosslinking had occurred through etherification in which the hydroxy groups were replaced by an ether linkage. The reduced possibility of conjugation with the ester carbonyl (conjugation lowers the electron density of the C=O bond thus decreasing its frequency), along with steric hindrances, increases the electron density and consequently shifts the carbonyl band to a higher frequency. As the epoxy ring reacts with the amine group from the AH or with the phenolic hydroxyl groups of TA and L, the intensity of this peak becomes lower in the cured samples of TA/L-based bio-epoxides (Figure 4 and Figure 5).

### 3.3. Tensile Sheer Strength Determination of the Adhesive Joint

**Figure 6:**
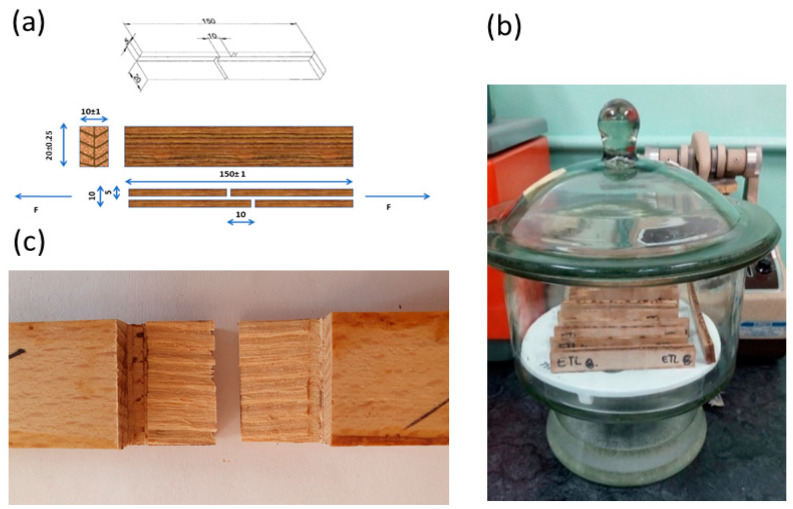
(**a**) preparing the samples for tensile shear strength determination; (**b**) samples prepared and conditioned for tensile shear strength determination; (**c**) joint sample after tensile shear testing that have undergone a 100% fracture through the wood.

The epoxy adhesives were prepared according to the formulations shown in Table 1. Firstly, the pure epoxy adhesive was prepared by blending the epoxy resin and the amine hardener at room temperature and at 120 °C. The other samples were also obtained by combining the corresponding epoxy (ER/EL) component and the hardener (AH/TA/L). Joint samples for adhesive testing were prepared according Figure 6a,b.

After the completion of the adhesion tests, the failure surface was analyzed using high-resolution imaging to estimate the type of failure. In addition to tensile shear strength, it is important to take into consideration the mode of failure during the mechanical test and the percentage of wood failure (Figure 6c). Depending on the relative strength of the adhesive to the adherend (wood) and the bond line quality, different failure modes can be observed: cohesive failure (both samples have an adhesive layer on the overlapping surfaces); adhesive failure (only one of the surfaces has adhesive); and adherend (wood) failure (breakage of the adherend itself so that the bonding surface is not altered) [7]. Thus, regarding wood adhesives, wood failure (Figure 6c) is preferred as the main aim is to obtain good bond line quality and high-performance wood adhesives. In that case, the wood failure percentage is usually used to estimate the adhesive/wood ratio that is removed from their surface contact. The adhesive bond of the highest quality must be stronger than the cohesive bond in the adhesive itself and the cohesive bond in the adherend-wood.

**Figure 7:**
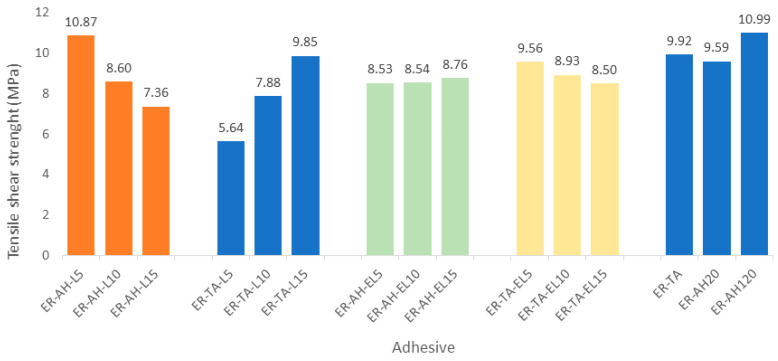
Tensile shear strength results for different adhesive blends.

The tensile shear strength of different samples of bio-epoxy wood adhesives based on lignin, epoxy lignin, and tannic acids were determined and compared, and the results are summarized in Table 2 and Figure 7. When it comes to wood adhesive, it is significant to efficiently evaluate the possibility of its application in wood adhesives. Additionally, it is also important to determine the adhesive properties, such as mechanical strength, water resistance, thermal, and rheological properties of the adhesives, and the possibility of penetration of the adhesive.

The crucial point in estimating the adhesive’s application potential is to apprehend the nature of the adhesive/wood interaction. Another important factor influencing the adhesive strength is connected to the extent of the adhesive’s contact with the adherend that depends on the possibility of adhesive penetration into the wood, thus enabling favorable contact of the adhesive with the wood fibers. For the utmost application of wood adhesives, water resistance is of crucial importance for their mechanical properties.

Epoxy resins consist of two components—an epoxy component and a hardener. Phenolic hydroxyls, such as the hydroxyls in lignin and tannic acid, are suitable for epoxy-polymerization reactions as the hardener replacement because of their hydroxyl-rich surfaces. The adhesive’s performance depends on the ratio of the epoxy component to the hardener, the particle size, the curing heat, and the curing time. Usually, in conventional epoxy chemistry, an equimolar ratio of epoxy groups to hardener is used. This study aimed to investigate a series of bio-based epoxy adhesives for wood and achieve a desirable adhesion strength by incorporating tannic acid, lignin, or epoxy lignin into commercial epoxy resin. Subsequently, the possibility of using lignin and tannic acids as a substitute for the amine hardener was examined, while the epoxy lignin represented a replacement for the epoxy component.

Examination of the failure of the samples showed that all samples had a 100% fracture through the wood. The commercial resin sample (ER-AH120) cured at higher temperatures showed a higher tensile shear strength (10.99 MPa vs. 9.59 MPa, Figure 7) as expected, since higher temperature and pressure have already proved to be beneficial for the crosslinking reaction [41]. This can be explained by the fact that the hot-pressing process facilitates the epoxy ring-opening reaction.

The first series was prepared by introducing lignin in amounts of 5%, 10% and 15%. The addition of lignin at a 5% blending ratio also showed an impressive tensile shear strength result of 10.87 MPa, similar to that of pure resin. This result indicated that the amount of 5% lignin introduced into the resin promoted the curing process and improved the tensile shear strength of the resulting adhesive. However, with increasing percentages of lignin, the tensile shear strength decreases. This effect is probably because lignin in higher amounts, as an insoluble material, brings about internal agglomerates which induce strong stress concentration effects and a phase separation between lignin and ER, which are the main reasons for the degradation of mechanical properties. Some authors also concluded that lignin, due to its polyphenolic structure, can replace the hardener because it can react with the epoxy resin [41]. However, not many studies were performed on the lignin being used in the application of epoxy resins. For example, Kong et al. [45] combined a conventional epoxy resin with a 5% hydrolyzed lignin and studied the curing kinetics and the bonding properties of the resins as adhesives. Yin et al. [34] investigated the mechanical properties and curing of an epoxy resin mixed with corn straw lignin obtained by enzymatic hydrolysis and concluded that the hydroxyl and carboxyl groups of the lignin could react with the epoxy groups, and that the polyphenol structure of the lignin could catalyze the curing reaction. Previous studies showed that the elongation or tensile strain increased significantly, but the tensile strength did not [46]. Lignin segments incorporated into the epoxy matrix induce the deformation of the crosslinked structure and increase the rigidity of the resin which leads to lower compatibility between the ER and the lignin. The next series was prepared with the aim of replacing the amine hardeners with tannic acids and lignin in different percentages. Surprisingly, the results showed an opposite trend from the previous ones. The higher the percentage of lignin, the higher the tensile shear strength value, from 5.64–9.85 MPa. Tannic acid (TA) possesses a hyperbranched biobased polyphenol/polyester moiety due to an abundance of reactive phenolic hydroxyl groups and can form highly crosslinked networks with epoxy resins. Additionally, owing to the similar structures of lignin and tannic acid, their higher compatibility reduces lignin agglomeration in the epoxy matrix, thus promoting its reaction with the epoxy component.

On the other side, some previous studies also demonstrated that lignin represents a favorable candidate for the replacement of the epoxy components in bio-epoxy resin production. Since lignin is an aromatic biopolymer comprising a variety of aliphatic and aromatic hydroxyl groups together with carboxylic functional groups, it can readily react with epichlorohydrin in order to obtain bio-epoxy resins based on epoxy lignin [39,45,47]. Besides improved reactivity, lignin epoxidation reduces its agglomeration and improves material flexibility. Hence, a series of samples were prepared in which epoxidized lignin (epoxy lignin) was introduced as a substitute for ER, however these adhesives exhibited somewhat lower tensile shear strength values ranging from 8.53–8.76 MPa (Figure 7), presumably because of the lower crosslinking capability of epoxy lignin compared to pure ER. This can also be confirmed with the higher tensile shear results of the adhesive series in which the amine hardener is replaced with tannic acids. These results also decrease with increasing epoxy lignin content indicating that tannin acids possess a greater potential for the crosslinking reaction with epoxy lignin than AH. The results also implied that tannin acids can effectively react with ER as well as EL [48]. The observed impressive adhesion strength (ER-TA, 9.92 MPa) is even higher than the tensile shear strength of the epoxy resin obtained with the commercial hardener at room temperature (ER-AH20, 9.59 MPa), and slightly lower than for the same at 120 °C (ER-TA120, 10.99). This effect can be explained by the fact that the high polar group content (including residual phenolic hydroxyl groups, ether linkages, and aromatic/alicyclic ester groups) not only reacts with ER, but also forms hydrogen bonds with wood lignocellulosic constituents and, therefore, induces strong adhesion bonds with wood surfaces.

### 3.4. SEM

**Figure 8:**
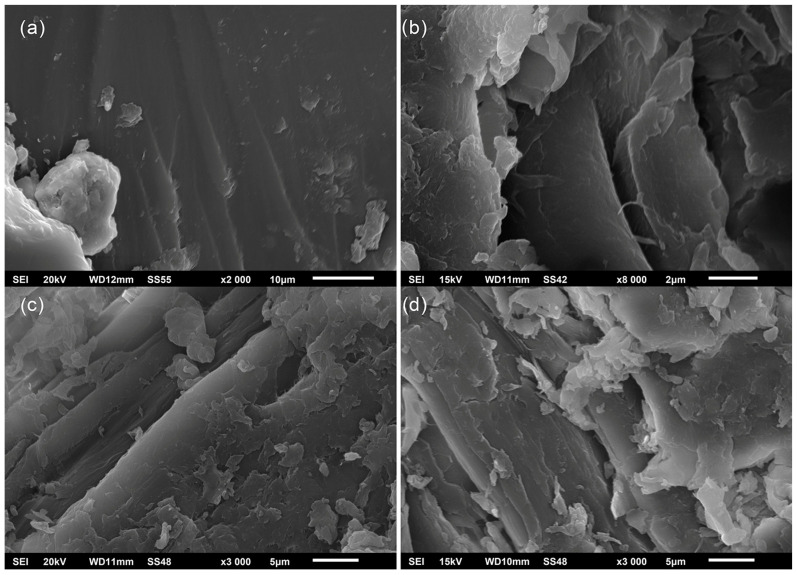
(**a**) ER-AH120; (**b**) ER-TA10; (**c**) ER-TA-EL10; and (**d**) ER-TA-L10.

The fractures in commercial epoxy adhesive and bio-epoxy adhesives in which the commercial AH was substituted with TA or lignin, or the ER was substituted with 10 wt% of epoxy functionalized lignin, were analyzed by SEM (Figure 8). The mode of failure is an important feature to take into consideration when discussing the strength of wood adhesives. Examination of the failure of the samples showed that all samples had a 100% fracture through the wood.

The fracture surfaces of the plywood after wet shear strength testing observed via SEM imaging showed that the fracture of the commercial epoxy adhesive is smooth with river-like patterns (lines), indicating a brittle failure mechanism (Figure 8a) [49]. The smoothness of the ER-TA adhesive’s fractured surface (Figure 8b) is slightly reduced indicating that the replacement of the AH with TA causes the appearance of a rougher fracture surface. However, phase separation is not observed in the ER-TA sample [49]. The opposite is found for the adhesive sample with 10 wt% of unmodified lignin (Figure 8d). After the addition of unmodified kraft lignin, it was observed that the fracture structure was much rougher compared to the commercial ER-AH120 adhesive. This can be explained by the lower amount of epoxy functionality in the investigated ER-TA-L10 adhesive and reduced cross-linking density which causes the formation of an irregular fracture surface. In order to improve the mechanical performance and increase the cross-linking density, epoxy modification of the lignin is performed. However, after adding 10 wt% of the epoxy-functionalized lignin (replacement for the ER), the fracture surface becomes more uniform (Figure 8c). This phenomenon indicates that the epoxy modification of lignin makes it more convenient for the bio-epoxy adhesive preparation because both phenolic hydroxyl and epoxy reactive groups participate in cross-linking [48] (Figure 5 and Figure 6).

## 4. Conclusions

New environmentally friendly wood construction materials also need innovative bio-based adhesives. There has been an increasing call for new bio-adhesives based on renewable sustainable resources to reduce negative environmental impacts. Bio-epoxy resins based on renewable resources such as natural polyphenols (lignin and tannic acids) offer huge potential in creating new polymeric materials. Herein, we reported a facile method of fabricating bio-based and high-performance epoxy thermosets through the curing of epoxy resin using TA and lignin as a substitute for the amine hardener, or epoxy lignin for the epoxy component.

This paper focused on the adhesive properties of bio-epoxy resins and the possibility of their application as wood adhesives. The curing reaction of epoxy resin with lignin/TA was confirmed via FTIR spectroscopy. Examination of the failure of the samples showed that all samples had a 100% fracture through the wood. All samples of bio-epoxy adhesives displayed significant tensile shear strength; the highest value was determined for ER-AH-L5, which decreased with an increasing percentage of lignin. A higher percentage of lignin leading to diminishing tensile shear strengths can be explained by the formation of lignin agglomerates.

Epoxy resins based on epoxy lignin as a substitute for DGEBA exhibited remarkably lower tensile shear strength values, presumably because of the lower crosslinking capability of epoxy lignin compared to pure ER. The addition of TA as a replacement for AH caused higher tensile shear strengths which decreased with increasing epoxy lignin content, leading to the conclusion that TA represents a promising replacement for amine hardeners due to its greater crosslinking potential with epoxy lignin than AH. This can be attributed to the high polar group content that can not only react with EL but also form hydrogen bonds with wood lignocellulosic to create strong adhesion bonds with wood surfaces.

Although novel bio-adhesives based on natural aromatic biopolymers are promising candidates for wood adhesives, taking into account their adhesive performance, the complete replacement of fossil fuel-based adhesives is still unattainable because of their low interaction with current synthetic adhesives that affect its adhesive performance. Chemical modification represents one of the possible solutions to make them more compatible.

## Figures and Tables

**Table 1:** Formulations of the prepared adhesives.

No.	Adhesive Formulation	Label	ER (g)	AH/TA (g)	L/EL (g)
1	CompA + CompB + L (5%)	ER-AH-L5	4	2	0.3
2	CompA + CompB + L (10%)	ER-AH-L10	4	2	0.6
3	CompA + CompB + L (15%)	ER-AH-L15	4	2	0.9
4	CompA + TA + L (5%)	ER-TA-L5	4	2	0.3
5	CompA + TA + L (10%)	ER-TA-L10	4	2	0.6
6	CompA + TA + L (15%)	ER-TA-L15	4	2	0.9
7	CompA + CompB + EL (5%)	ER-AH-EL5	4	2	0.3
8	CompA + CompB + EL (10%)	ER-AH-EL10	4	2	0.6
9	CompA + CompB + EL (15%)	ER-AH-EL15	4	2	0.9
10	CompA + TA + EL (5%)	ER-TA-EL5	4	2	0.3
11	CompA + TA + EL (10%)	ER-TA-EL10	4	2	0.6
12	CompA + TA + EL (15%)	ER-TA-EL15	4	2	0.9
13	CompA + CompB	ER-AH20	4	2	-
14	CompA + CompB	ER-AH120	4	2	-
15	CompA + TA	ER-TA	4	2	-

**Table 2:** Tensile shear strength results.

**No**	**Label**	**MPa**
1	ER-AH-L5	10.87 ± 0.37
2	ER-AH-L10	8.60 ± 0.25
3	ER-AH-L15	7.36 ± 0.22
4	ER-TA-L5	5.64 ± 0.17
5	ER-TA-L10	7.88 ± 0.27
6	ER-TA-L15	9.85 ± 0.33
7	ER-AH-EL5	8.53 ± 0.18
8	ER-AH-EL10	8.54 ± 0.22
9	ER-AH-EL15	8.76 ± 0.16
10	ER-TA-EL5	9.56 ± 0.32
11	ER-TA-EL10	8.93 ± 0.25
12	ER-TA-EL15	8.50 ± 0.14
13	ER-AH20	9.59 ± 0.15
14	ER-AH120	10.99 ± 0.30
15	ER-TA	9.92 ± 0.21

## Data Availability

The original contributions presented in the study are included in the article, further inquiries can be directed to the corresponding authors.
